# Caspase-Dependent and Caspase-Independent Pathways Are Involved in Cadmium-Induced Apoptosis in Primary Rat Proximal Tubular Cell Culture

**DOI:** 10.1371/journal.pone.0166823

**Published:** 2016-11-18

**Authors:** Gang Liu, Hui Zou, Tongwang Luo, Mengfei Long, Jianchun Bian, Xuezhong Liu, Jianhong Gu, Yan Yuan, Ruilong Song, Yi Wang, Jiaqiao Zhu, Zongping Liu

**Affiliations:** College of Veterinary Medicine, Yangzhou University, and Jiangsu Co-innovation Center for Prevention and Control of Important Animal Infectious Diseases and Zoonoses, and Jiangsu Key Laboratory of Zoonosis, Yangzhou, Jiangsu, PR China; University of Manitoba, CANADA

## Abstract

We designed this study to investigate whether cadmium induces caspase-independent apoptosis and to investigate the relationship between the caspase-dependent and caspase-independent apoptotic pathways. Cadmium (1.25–2.5 μM) induced oxidative stress in rat proximal tubular (rPT) cells, as seen in the reactive oxygen species levels; N-acetylcysteine prevented this. Cyclosporin A (CsA) prevented mitochondrial permeability transition pore opening and apoptosis; there was mitochondrial ultrastructural disruption, mitochondrial cytochrome c (cyt c) translocation to the cytoplasm, and subsequent caspase-9 and caspase-3 activation. Z-VAD-FMK prevented caspase-3 activation and apoptosis and decreased BNIP-3 (Bcl-2/adenovirus E1B 19-kDa interacting protein 3) expression levels and apoptosis-inducing factor/endonuclease G (AIF/Endo G) translocation. Simultaneously, cadmium induced prominent BNIP-3 expression in the mitochondria and cytoplasmic AIF/Endo G translocation to the nucleus. BNIP-3 silencing significantly prevented AIF and Endo G translocation and decreased the apoptosis rate, cyt c release, and caspase-9 and caspase-3 activation. These results suggest that BNIP-3 is involved in the caspase-independent apoptotic pathway and is located upstream of AIF/Endo G; both the caspase-dependent and caspase-independent pathways are involved in cadmium-induced rPT cell apoptosis and act synergistically.

## 1. Introduction

Cadmium is gaining attention as a known occupational hazard and environmental pollutant that can cause a series of biochemical and physiological dysfunctions in humans. The exposure routes have principally been contact with batteries, paints, fertilizers, and automobiles. As with other complex organic pollutants, microorganisms cannot degrade cadmium. Cadmium accumulates in the ecosystem and enters the food chain through contaminated water and soil and has an extremely long biological half-life. As a multi-organ toxicant, cadmium exerts toxic effects on the brain, liver, kidney, heart, and bone [1]. The kidney is the primary site for the initial accumulation of cadmium, and the proximal tubule cells are sensitive to cadmium-induced damage [2].

The mitochondria play a central role in regulating apoptotic cell death. Numerous pro-apoptotic factors and damage pathways act on the mitochondria to induce oxidative stress, and reactive oxygen species (ROS) overproduction can directly result in mitochondrial permeability transition pore (MPTP) opening, followed by mitochondrial release of apoptogenic signaling molecules, such as procaspases, cytochrome c (cyt c), apoptosis-inducing factor (AIF), and endonuclease G (Endo G) [3, 4]. Cadmium-induced apoptosis occurs mostly via activation of the mitochondrial apoptotic pathways [5, 6].

The apoptogenic potential of cadmium on cells and primary rat kidney cell culture has been reported [7–10]. Previously, we showed that lead induces oxidative stress in rat proximal tubular (rPT) cells and resulted in apoptosis through MPTP opening [11]. ROS enhancement in murine splenocytes and thymocytes induces mitochondrial membrane depolarization, which leads to caspase-3 activation and DNA fragmentation [12, 13].

Many studies have also focused on the caspase-independent apoptotic pathway, known as the AIF/Endo G pathway. Caspase-independent apoptosis is activated by BNIP-3 (Bcl-2/adenovirus E1B 19-kDa interacting protein 3), which induces mitochondrial AIF release; Endo G acts as a modulator. Forced BNIP-3 expression by plasmid transfection results in mitochondrial Endo G release and nuclear translocation [14]. BH3 domain of BNIP-3 interacted with anti-apoptotic protein to form dimers, which was able to promote the apoptosis and the homodimerization of TM domain also promoted apoptosis. The investigation confirmed that homodimerization of BNIP-3’s TM domain involved in mitochondria apoptosis pathway [15]. While there was no evidence for homodimerization of TM domain involved in caspase-independent apoptosis pathway. Overexpression of BNIP-3, an upstream effector of AIF, induces MPT and cyt c release; BNIP-3 silencing by short hairpin RNA (shRNA) increases mitochondrial cyt c levels and blocks the caspase-dependent apoptotic pathway [16]. BNIP-3 located in different positions in cells. According to studies, BNIP3 was involved in promoting apoptosis mainly engaged in mitochondria, it could bind to mitochondria and make the mitochondrial dysfunction. While, BNIP3 bound to the promoter of the AIF gene and represses its expression when it translocated to nuclei. BNIP3-mediated reduction in AIF expression leads to decreased temozolomide-induced apoptosis in glioma cells and transcriptional repression function for BNIP3 causing reduced AIF expression and increased resistance to apoptosis [17]. BNIP-3 also involved in autophagy induction. BNIP-3's transmembrane domain that preserve mitochondrial localization, but disrupt dimerization fail to induce autophagy [18]. BNIP-3 dimerization is thought to free Beclin-1 from its interaction with anti-apoptotic Bcl-2 family proteins, then to cause autophagy [19]. Although the caspase-dependent and caspase-independent apoptotic pathways are separate, there is evidence of crosstalk between the two [20]. Furthermore, caspase inhibitors such as Z-VAD-FMK prevent mitochondrial AIF release [20–23].

We aimed to identify the role of the caspase-dependent and caspase-independent pathways in cadmium-induced apoptosis and the relationship between the two in rPT cells. We found that both pathways are involved in cadmium-induced rPT cell apoptosis and affect each other.

## 2. Materials and Methods

### 2.1. Animals and treatment

The Sprague-Dawley rats weighing between 180 g and 200 g were obtained from the Comparative Medicine Centre of Yangzhou University (Yangzhou, China). The animals were housed individually on a 12 h light/dark cycle with unlimited standard rat food and double distilled water (DDW). All experimental procedures were conducted in accordance with the recommendations in the Guide for the Care and Use of Laboratory Animals of the National Research Council and were approved by the Animal Care and Use Committee of Yangzhou University (Approval ID: SYXK (Su) 2007–0005). All surgeries operations were performed under sodium pentobarbital anesthesia, and all efforts were made to minimize any suffering experienced by the animals used in this study.

### 2.2. Reagents

All chemicals were of the highest-grade purity available. Dulbecco’s modified Eagle’s medium (DMEM)-F12 (1:1), fetal bovine serum (FBS), trypsin-EDTA, and collagenase IV were from Gibco (Grand Island, NY, USA). Cadmium acetate, calcein acetoxymethyl ester (calcein-AM), cobalt chloride (CoCl_2_), 2’, 7’-dichlorofluorescein diacetate (DCFH-DA), and DAPI were from Sigma-Aldrich (St. Louis, MO, USA). Cell Counting Kit-8 (CCK-8) was from Dojindo Laboratories (Tokyo, Japan). The mitochondria isolation kit for cultured cells was from Pierce Biotechnology (Rockford, IL, USA).

The following primary antibodies were used: anti–cyt c (CST, #14940), anti–cyt c oxidase subunit IV (COX IV) (CST, #11967), anti–cleaved caspase-9 (CST, #9506), anti–cleaved caspase-3 (CST, #9664), anti–β-actin (CST, #4970S) were from Cell Signaling Technology (Boston, USA); anti–BNIP-3 (Abcam, ab 109362), anti-AIF (Abcam, ab1998), anti–Endo G (Abcam, ab9647), and anti–lamin B1 (Abcam, ab16048) were from Abcam (Cambridge, USA). All secondary antibodies were from Beijing Zhongshan Golden Bridge Biotechnology (Beijing, China). The PrimeScript RT reagent kit with gDNA Eraser and SYBR Premix Ex Taq RT-PCR kit were from Takara (Dalian, China). Accutase cell detachment solution and the annexin V–fluorescein isothiocyanate/propidium iodide (FITC/PI) apoptosis detection kit were from Becton-Dickinson (San Diego, CA, USA). All other chemicals were from Sigma-Aldrich.

### 2.3. Cell culture and cadmium exposure conditions

The rPT cells were obtained from the kidneys of Sprague-Dawley rats (from the Comparative Medicine Centre of Yangzhou University) with body weights between 180 g and 200 g. Intraperitoneal injection of sodium pentobarbital (2%, 0.31 ml/100 g) to anesthetize rats. Breaking the neck to death 5 min latter when the rats were in a deep coma. The rats were transferred to super-clean worktable after 75% alcohol soak for 2 minutes. Then, opened the abdominal cavity and removed kidneys of rats under aseptic conditions. rPT cell isolation, identification, and culture were performed as previously described [24]. Primary cells and subcultures were cultured in DMEM/F12 supplemented with 15% FBS, 0.25 g/L glutamine, 100 U/mL penicillin, and 100 μg/mL streptomycin at 37°C in 95% air and 5% CO_2_. rPT cell identity was confirmed by alkaline phosphatase antibody staining against specific proximal tubular antigens. The purity of the isolated primary rPT cells was >95%; the cells were subcultured using trypsin-EDTA digestion. Cells cultured for 12 h had the highest viability (according to the growth curve, data not shown). Based on the doses in a previous study [25], cells were treated with 1.25, 2.5, or 5.0 μM cadmium; the cadmium acetate stock solution was dissolved in sterile ultrapure water.

### 2.4. N-acetylcysteine (NAC) and Cyclosporin A (CsA) treatment

rPT cells were seeded in 6-well plates and pretreated 30 min with NAC or CsA before Cd treatment when the cell fusion rate was 60–70%. NAC or CsA were freshly prepared in deionized water and filter-sterilized before use, the pH of the NAC or CsA was adjusted to 7.0.

### 2.5. DAPI staining

Apoptotic morphological changes in the nuclei were detected by staining with DAPI (4',6-diamidino-2-phenylindole). rPT cells (2 × 10^5^ cells per well) were seeded onto sterile cover slips in 24-well plates. After 12-h treatment with 0, 1.25, 2.5, or 5.0 μM cadmium, the medium was removed. The cells were washed with ice-cold phosphate-buffered saline (PBS), fixed with paraformaldehyde (4% w/v) for 10 min at room temperature, and incubated with DAPI staining solution (50 mM in PBS) for 10 min in the dark. After washing in PBS three times, the cells were viewed under a Leica inverted fluorescence microscope (Wetzlar, GER) at an excitation wavelength of 352 nm. To assess the extent of cadmium-induced apoptosis, 200 cells per experiment were randomly selected and the apoptotic cells therein were counted; each experiment was performed in triplicate.

### 2.6. Measurement of MPTP activity

MPTP opening in rPT cells was detected using calcein-AM and CoCl_2_ loading, resulting in mitochondrial localization of calcein fluorescence [26]; these reagents were used to monitor the MPTP activity. rPT cells (2 × 10^5^ cells per well) were seeded onto sterile cover slips in 24-well plates, loaded for 30 min at 37°C with 2 μM calcein-AM, followed by 1-h incubation with 2 mM CoCl_2_ after 12-h incubation with 2.5 μM cadmium, and then washed twice with PBS. The cover slips were fixed with paraformaldehyde (4% w/v) for 10 min at room temperature and then imaged under a laser scanning confocal microscope (LSM 710; Zeiss, Jena, Germany). The change in fluorescence intensity was measured with excitation at 488 nm and emission at 525 nm.

### 2.7. Flow cytometry analysis

All subsequent assays were carried out on a Beckman Coulter fluorescence-activated cell sorter (CyAn ADP 7; Brea, CA, USA). rPT cells were seeded in 6-well plates and treated with 0, 1.25, 2.5, or 5.0 μM cadmium for 12 h when the cell fusion rate was 60–70%. Subsequently, the adherent cells were collected with the Accutase cell detachment solution by 5-min centrifugation at 1500 rpm. Each treatment group yielded at least 1.5 × 10^6^ cells, which were washed twice with PBS and incubated with fluorescent dyes for the flow cytometric analysis.

### 2.8. Detection of apoptosis

Apoptotic cells were evaluated using annexin V–FITC/PI staining. The total apoptotic proportion is presented as the sum of early and late apoptotic cells, which was determined as the percentage of annexin V^+^/PI^-^ and annexin V^+^/PI^+^ cells, respectively. After 12-h staining, the harvested cells were labeled with annexin V–FITC and PI according to the manufacturer’s protocol. FITC and PI fluorescence was characterized using an FL-1 filter (530 nm) and FL-2 filter (585 nm), respectively; 10,000 events were acquired.

### 2.9. ROS measurement

Intracellular ROS were determined using flow cytometry and DCFH-DA staining. DCFH-DA can be cleaved to form non-fluorescent dichlorofluorescein (DCFH) in the cells and is oxidized to fluorescent dichlorofluorescein (DCF) by ROS. Cells (1.5 × 10^6^) were incubated with 100 μM DCFH-DA at 37°C for 30 min, washed twice with PBS, and the fluorescence intensity (FL-1, 530 nm) of 10,000 cells was measured using a flow cytometer.

### 2.10. Cell fraction preparation

After 12-h treatment with 0, 1.25, 2.5, or 5.0 μM cadmium, cells were harvested by Accutase™ Cell Detachment Solution and washed twice with PBS. To obtain the mitochondrial and cytosolic protein extracts, the harvested cells were subfractionized in homogenization buffer. The mitochondrial and cytosolic fractions were isolated with the method described by Jayanthi et al. [27]. The pellet and supernatant contained the mitochondrial fraction and cytosolic fraction, respectively.

### 2.11. Western blot analysis

After protein quantification with a bicinchoninic acid (BCA) protein assay kit (Beyotime, Shanghai, China), equal amounts of protein were separated by 8–15% sodium dodecyl sulfate–polyacrylamide gel electrophoresis and transferred to 0.22-μm or 0.45-μm polyvinylidene difluoride membranes followed by blocking in 5% skim milk for 1 h at room temperature. The membranes were incubated overnight at 4°C with the following primary antibodies: anti–cyt c (1:1000), anti–COX IV (1:1000), anti–cleaved caspase-9 (1:1000), anti–cleaved caspase-3 (1:1000), anti-AIF (1:1000), anti–BNIP-3 (1:1000), anti–Endo G (1:1000), and anti–β-actin (1:1000), and then with the appropriate secondary antibodies (1:5000) and enhanced chemiluminescence reagent. Each test was performed in three experiments with different batches of cells. Protein levels were determined by computer-assisted densitometric analysis (GS-800 densitometer, Quantity One; Bio-Rad). The band volumes were determined by standard scanning densitometry with normalization of densitometry measures to β-actin or COX IV.

### 2.12. Immunofluorescence assays

rPT cells (2 × 10^5^ cells per well) were seeded onto sterile cover slips in 24-well plates and treated with 0, 1.25, 2.5, or 5.0 μM cadmium for 12 h; there were three replicates per group. Next, the cells were washed twice with PBS, and fixed on the coverslips with 4% paraformaldehyde. Then, the monolayer was permeated with 0.5% Triton X-100 and the cells were blocked with 5% bovine serum albumin (BSA). The cells were incubated with anti-AIF antibody (1:100) overnight at 4°C, washed with PBS, stained with Alexa Fluor 488–labeled goat anti-rabbit immunoglobulin G (IgG) (H+L) (1:500) for 1 h at room temperature, and the nuclei were stained with DAPI (5 μg/mL) in the dark. A Leica inverted phase contrast microscope (Wetzlar, GER) was used to examine and analyze the transfer rate of AIF into the nuclei.

### 2.13. Statistical Analysis

Data from the present study are presented as mean±SD from at least three independent experiments with different batches of cells, and each one was performed in duplicate or triplicate. Statistical comparisons were made using one-way analysis of variance (ANOVA) (Scheffe’s F test) after ascertaining the homogeneity of variance between the treatments. All statistical data were analyzed using SPSS 19.0 (SPSS, Chicago, IL, USA). The critical value for statistical significance was *P<*0.05.

## 3. Results

### 3.1. Cadmium induces mitochondrial oxidative stress and dysfunction in rPT cells

**Fig 1:**
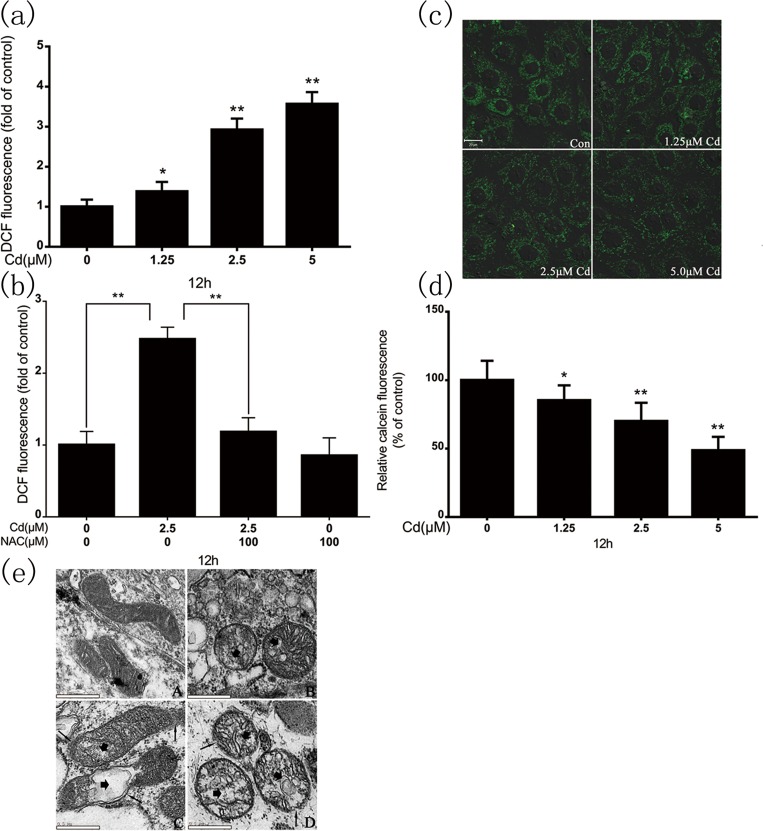
(a) Cadmium induced ROS generation dose-dependently in rPT cells. (b) Intracellular ROS levels in rPT cells after 12-h cadmium (2.5 μM) treatment in the absence or presence of NAC (100 μM). DCF fluorescence was measured using a flow cytometer with FL-1 filter. Confocal microscopy of cadmium-induced MPTP opening. (c) Representative confocal images of cadmium-induced rPT cells. (d) Quantification of calcein fluorescence. Calcein fluorescence values were quantified relative to the control, where the fluorescence value was set at 100%. (e) Representative electron micrographs of rPT cell mitochondria following cadmium exposure (×6600 magnification). Figure shows membrane disruption (thin arrows), swelling, and damaged cristae (thick arrows). Fluorescence results are expressed as mean fluorescence intensity, and are the mean ± SD of three separate experiments, each performed in triplicate (n = 9). *P < 0.05, **P < 0.01 as compared to control.

We detected intracellular superoxide accumulation in cadmium-treated rPT cells. The cells were stained with DCFH-DA, a superoxide-specific dye, after 12-h cadmium treatment (Fig 1A). Cadmium significantly increased the intracellular superoxide levels, which co-treatment with 100μM N-acetylcysteine (NAC) eliminated (Fig 1B). Next, we examined the changes in MPTP opening and mitochondrial morphology following cadmium treatment. Fig 1C and 1D shows that reduced mitochondrial calcein fluorescence represented MPTP opening and it was dose-dependent during cadmium exposure. The cadmium-induced changes in mitochondrial morphology were assessed by transmission electron microscopy. The morphological changes were typical of mitochondrial damage, i.e., swelling, rupture of the outer membrane, and distorted cristae (disruption or loss); the severity of the damage increased with the cadmium dose (Fig 1E).

### 3.2. Effect of CsA on MPTP and apoptosis

**Fig 2:**
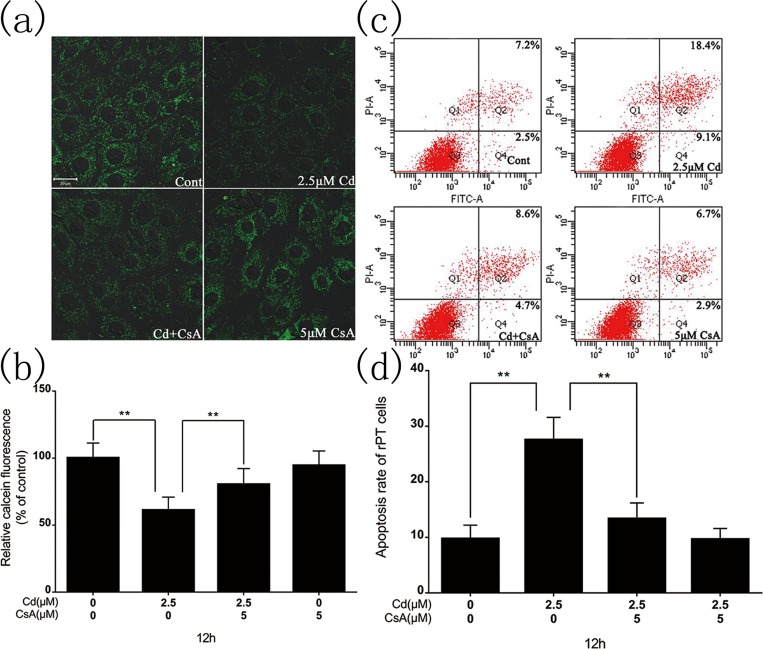
Confocal microscopy of MPTP opening after 12-h cadmium (2.5 μM) treatment in the absence or presence of CsA (5 μM). (a) Representative confocal images of cadmium-induced rPT cells. (b) Quantification of calcein fluorescence. The calcein fluorescence values were quantified relative to the control, where the fluorescence value was set at 100%. (c) Flow cytometry detection of the apoptosis rate after 12-h cadmium (2.5 μM) treatment in the absence or presence of CsA (5 μM) with annexin V–FITC/PI staining. (d) Percentage of apoptotic cells. Results are the mean ± SD of three separate experiments, each performed in triplicate (n = 9). **P < 0.01 as compared to control.

Fig 2A and 2B show that co-incubation with CsA (an MPTP inhibitor) significantly reversed cadmium-mediated MPTP opening. The mitochondrial calcein fluorescence drastically increased from 35.7% (2.5 μM cadmium alone) to 83.6% (cadmium+CsA). However, CsA alone had no effect on MPTP opening. Annexin V/PI staining was used to determine the apoptotic cells after 12-h cadmium exposure. Fig 2C and 2D show that 2.5 μM cadmium significantly enhanced the number of apoptotic cells (early and late), being 2.84-fold that of the control. CsA co-treatment significantly prevented apoptosis in the treated cells, being 1.37-fold that of the control.

### 3.3. Cyt c release and caspase-9 and caspase-3 activation as a measure of the caspase-dependent apoptotic pathway

**Fig 3:**
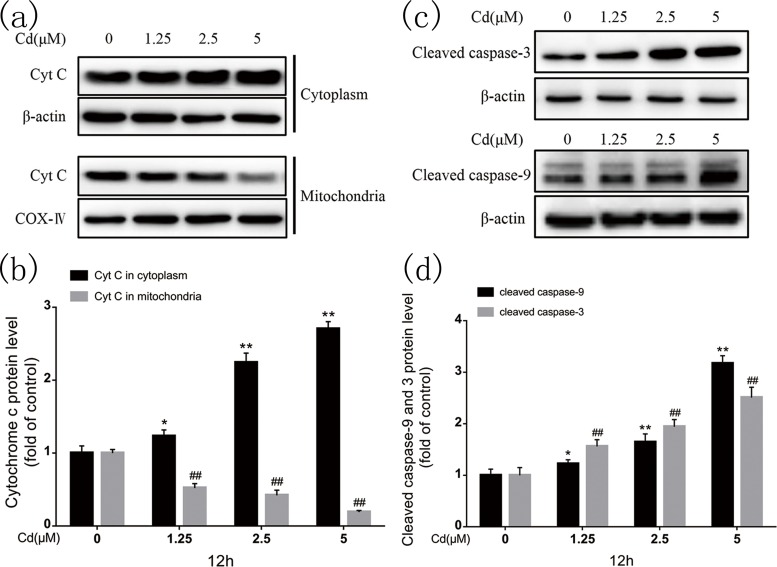
Cadmium induced mitochondrial cyt c release to the cytoplasm and subsequent caspase-9 and caspase-3 activation in rPT cells. (a, c) Representative western blots of cyt c, cleaved caspase-9, and cleaved caspase-3. (b, d) Quantitative analysis of cyt c, cleaved caspase-9, and cleaved caspase-3 western blots; grayscale of the control was set at 1. Quantitative analysis was performed with images from three independent experiments (mean ± SD, n = 3). *P < 0.05, **P < 0.01, and ^*##*^*P* < 0.01 as compared to control.

Fig 3A show that immunoblotting indicated significant mitochondrial cyt c release to the cytoplasm after 12-h cadmium exposure. In addition, quantification (Fig 3B) demonstrated that cadmium induced cyt c release dose-dependently. Cyt c released into the cytoplasm activates caspase-9. Cadmium increased cleaved caspase-9 protein expression dose-dependently; Cleaved caspase-3 is an execution protein in apoptosis; cadmium also increased its expression dose-dependently (Fig 3C and 3D). These results confirm that the caspase-dependent pathway is involved in cadmium-induced apoptosis in rPT cells.

### 3.4. BNIP-3 is involved in the caspase-independent apoptotic pathway and induces mitochondrial AIF and Endo G nuclear translocation

**Fig 4:**
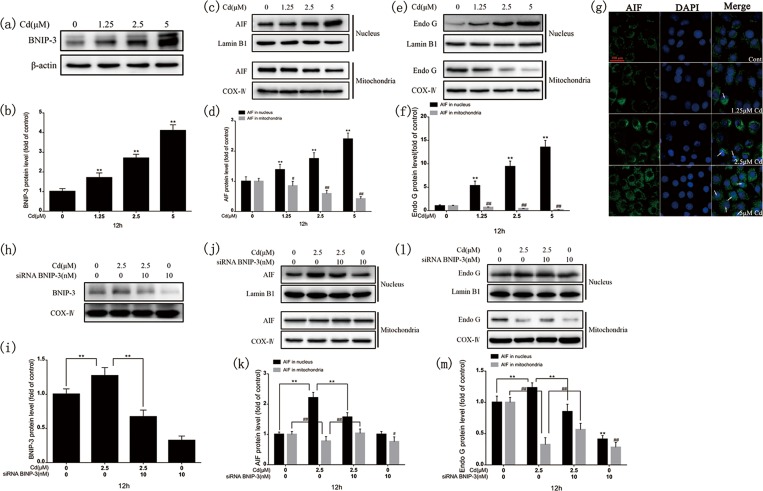
Cadmium induced BNIP-3 expression and cytoplasmic AIF and Endo G translocation to the nucleus after 12-h cadmium treatment. (a, c, e) Representative images of BNIP-3, AIF, and Endo G western blots. (b, d, f) Quantitative analysis of BNIP-3, AIF, and Endo G; grayscale of the control was set at 1. (g) Cadmium treatment (12 h) triggered AIF nuclear translocation dose-dependently. rPT cells were stained with anti-AIF antibodies and Alexa Fluor 488–labeled goat anti-rabbit IgG. AIF nuclear translocation was evaluated under fluorescence microscopy with DAPI staining. Scale bar: 50 μm. Effects of BNIP-3 silencing on changes in BNIP-3 expression and cytoplasmic AIF and Endo G translocation to the nucleus after 12-h cadmium treatment in the absence or presence of BNIP-3 small interfering RNA (siRNA). (h, j, l) Representative images of BNIP-3, AIF, and Endo G western blots. (i, k, m) Quantitative analysis of BNIP-3, AIF, and Endo G; grayscale of the control was set at 1. Results are from three independent experiments (mean ± SD, n = 3). **P < 0.01, ^*#*^*P* < 0.05, and ^*##*^*P* < 0.01 as compared to control.

Immunoblotting demonstrated that BNIP-3 protein levels were increased, as was mitochondrial AIF and Endo G translocation to the nucleus, dose-dependently after 12-h cadmium exposure (Fig 4A–4F). Fig 4G shows that AIF staining exhibited a granular pattern in the cytosol of the control group and was restricted mainly to the nucleus, as indicated by colocalization with DAPI labeling after 12-h cadmium exposure. Immunoblotting showed that mitochondrial AIF and Endo G translocation to the nucleus was decreased after BNIP-3 knockdown (Fig 4H–4M). The results confirm that BNIP-3 is involved in the caspase-independent apoptotic pathway and causes AIF and Endo G nuclear translocation.

### 3.5. Influence of caspase inhibitor on the caspase-independent apoptotic pathway

**Fig 5:**
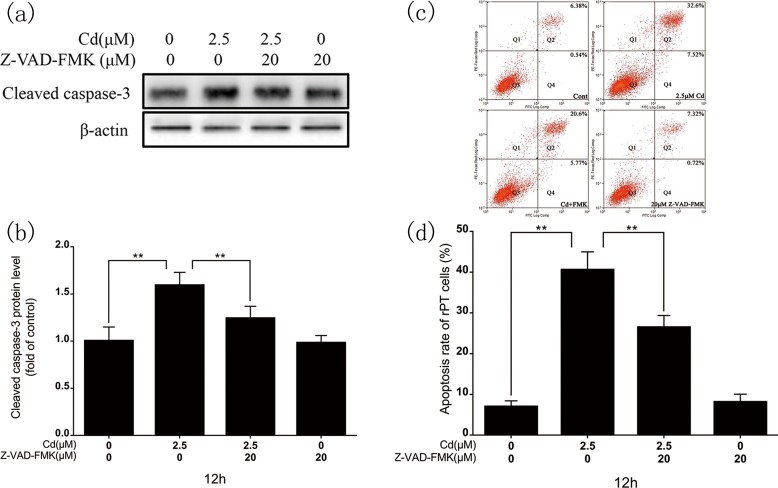
Effects of Z-VAD-FMK on cadmium-induced rPT cell apoptosis. (a) Representative images of cleaved caspase-3 western blot after 12-h cadmium treatment in the absence or presence of Z-VAD-FMK. (b) Quantitative analysis of cleaved caspase-3; grayscale of the control was set at 1. (c) Flow cytometry assessment of the rPT cell apoptosis rate after 12-h cadmium treatment with/without Z-VAD-FMK. (d) Percentage of apoptotic cells. Results are expressed as the mean ± SD of three separate experiments, each performed in triplicate (n = 9). **P < 0.01 as compared to control.

**Fig 6:**
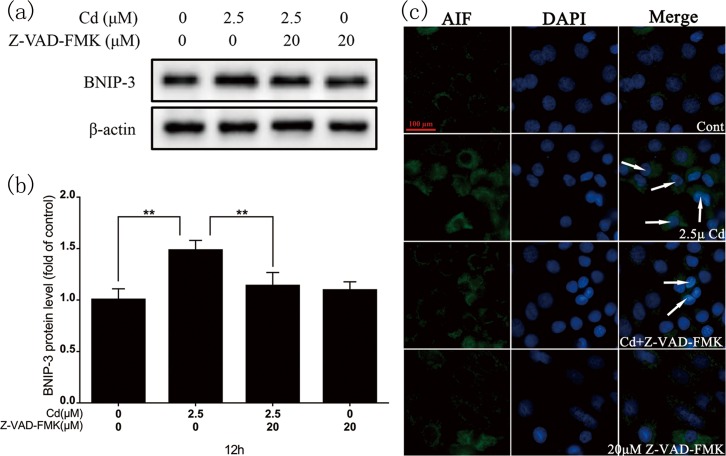
Effect of Z-VAD-FMK on BNIP-3 expression and cytoplasmic AIF translocation to the nucleus after 12-h cadmium treatment. (a) Representative images of BNIP-3 western blot. (b) Quantitative analysis of BNIP-3; grayscale of the control was set at 1. (c) Twelve-hour cadmium treatment with/without Z-VAD-FMK triggered AIF nuclear translocation. rPT cells were stained with anti-AIF antibodies and Alexa Fluor 488–labeled goat anti-rabbit IgG. AIF nuclear translocation was evaluated under fluorescence microscopy with DAPI staining. Scale bar: 50 μm. Results are from three independent experiments (mean ± SD, n = 3). **P < 0.01 as compared to control.

Cadmium (2.5 μM) and Z-VAD-FMK (20 μM, a caspase inhibitor) co-treatment decreased cleaved caspase-3 protein expression (Fig 5A and 5B). Flow cytometry revealed a significantly decreased apoptotic rate in the co-treatment group (Fig 5C and 5D). Immunoblotting showed that BNIP-3 protein levels (Fig 6A and 6B) and AIF transfer levels (Fig 6C) in the co-treatment group were significantly decreased compared with the cadmium-only group.

### 3.6. Influence of BNIP-3 silencing on the caspase-dependent apoptotic pathway

**Fig 7:**
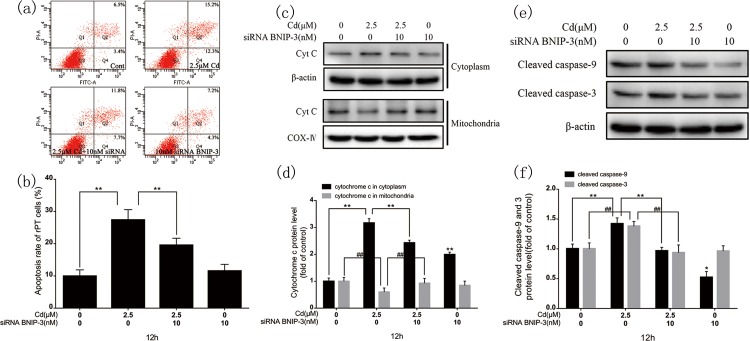
Effects of BNIP-3 silencing on cadmium-induced apoptosis, mitochondrial cyt c release to the cytoplasm, and changes in caspase-9 and caspase-3 expression. (a) Flow cytometry assessment of the rPT cell apoptosis rate after 12-h cadmium and/or BNIP-3 siRNA treatment. (b) Percentage of apoptotic cells. (c, e) Representative images of cyt c, caspase-9, and caspase-3 western blots. (d, f) Quantitative analysis of cyt c, caspase-9, and caspase-3; grayscale of the control was set at 1. Results are from three independent experiments (mean ± SEM, n = 3). *P < 0.05, **P < 0.01, and ^*##*^*P* < 0.01 as compared to control.

The earlier results confirmed that BNIP-3 is involved in the caspase-independent apoptotic pathway and induces mitochondrial AIF and Endo G translocation to the nucleus. BNIP-3 silencing blocked the caspase-independent pathway in cadmium-induced apoptosis, and clearly decreased the apoptotic rate (Fig 7A and 7B). Immunoblotting showed significantly decreased mitochondrial cyt c release to the cytoplasm after BNIP-3 knockdown (Fig 7C and 7D) and decreased cleaved caspase-9 and caspase-3 expression levels (Fig 7E and 7F).

## 4. Discussion

Using rPT cells as an in vitro model, we demonstrate that cadmium preferentially induces mitochondrial oxidative stress, dysfunction, MPT, and apoptosis. Oxidative stress promotes apoptosis in primary rPT cell cultures exposed to cadmium [5, 25] and exploring the role of the mitochondrial apoptosis pathway, the caspase-dependent and caspase-independent apoptotic pathways, and the relationship between the two in rPT cell apoptosis, is necessary.

Cadmium induces rPT cell apoptosis, in which oxidative stress plays a pivotal role [25]. Our results showed that cadmium-induced preferential mitochondrial superoxide accumulation leads to mitochondrial dysfunction, which NAC prevented. ROS can directly result in MPTP opening, which facilitates MPT induction [3], the closing or opening state of MPTP, enabling tight regulation of mitochondria-mediated apoptosis [28]. Our results reveal that cadmium can lead to morphological changes typical of mitochondrial damage, including matrix swelling, outer membrane rupture, and distorted cristae. Cadmium also induced MPTP opening, triggering the release of apoptogenic proteins into the cytosol, which CsA prevented. In the cadmium and CsA co-treatment group, the rate of rPT cell apoptosis was prevented partially as compared to the cadmium-only group. Therefore, the cadmium-induced oxidative stress–induced MPTP opening, which triggers apoptogenic factor release, was halted.

The release of mitochondrial pro-apoptotic proteins such as cyt c (caspase-dependent), and AIF and Endo G (caspase-independent) strengthened the occurrence of multiple apoptotic pathways. Caspase-9 activation subsequently activates caspase-3, and requires cyt c for apoptosome formation [29, 30]. We found that oxidative stress induced cyt c release and caspase-9 and caspase-3 activation, and ultimately led to apoptosis, which is in accordance with previous research on different cell types [31–34]. A similar study indicated that the use of 10 μmol/L of Cd induces cyt c release after 24 hours of Cd treatment [35]. Compared with our results, they used a lower dose and shorter time that induced cyt c release may due to primarily cultured cells was more sensitive to Cd. Intracellular zinc (Zn) depletion also induce apoptosis and shown as loss of ΔΨ, release of cyt c and activation of caspase-9 and caspase-3 [36]. This similar phenomenon may due to Zn from the zinc enzyme could be replaced by Cd and loss of function. Meanwhile, Zn is a part of the antioxidant defence system. Zn depletion will lead to oxidative stress which also induced by Cd.

AIF is a mitochondrial protein that translocates to the cytosol and the nucleus, mediating caspase-independent apoptosis in a number of model systems [37–40]. Endo G participates in mitochondrial DNA copying, recombination, and repair [41]; induced by oxidative stress, it translocates from the mitochondria to the nucleus [42]. Endo G released from the mitochondria interacts with AIF in the nucleus and is involved in caspase-independent apoptosis in *Caenorhabditis elegans* [43, 44]; in neurodegenerative disease, both Endo G and AIF expression levels are decreased in the mitochondria but are increased in the nuclei [45]. AIF cannot cut DNA; it is possible that both Endo G and AIF are involved in nuclear DNA degradation [44, 46]. BNIP-3 is a BH3-only pro-apoptotic member of the Bcl-2 family: it mediates cell death via different pathways, including the mitochondrial pathway [47]. Endoplasmic reticulum–targeted BNIP-3 induces cell death that the anti-apoptotic protein Bcl-2 can block. Mitochondria-targeted BNIP-3 initiates apoptosis, inducing MPT and mitochondrial membrane potential dissipation [48], and Bcl-2 expression cannot prevent it [47]. BNIP-3 and AIF cooperate to induce apoptosis and cavitation in epithelial morphogenesis [16]. Meanwhile, silencing BNIP-3 prevents Endo G translocation and DNA degradation [49]. We found that BNIP-3 protein levels and AIF and Endo G translocation were increased during cadmium-induced rPT cell apoptosis, while BNIP-3 silencing decreased AIF and Endo G translocation. In short, BNIP-3 is involved in the caspase-independent apoptotic pathway and it is located upstream of AIF/Endo G.

Under different stimuli, PARP-1 [poly (ADP-ribose) polymerase-1] activation triggers mitochondrial AIF release and translocation to the nucleus [50–52]. PARP-1 activation produces PAR in the nucleus, which is released into the cytosol and colocalizes with the mitochondria to induce AIF release [53]. The mature AIF is loosely bound on the mitochondrial outer membrane [54], from which PAR can detach it [55]. Others have reported that without caspase activity, AIF/Endo G still translocates to the nucleus under cadmium induction [6, 56]. Consequently, the relationship between the caspase-dependent and caspase-independent apoptotic pathways is controversial due to the uncertainty as to whether there is a site upstream of AIF/Endo G. We show that BNIP-3 is involved in the caspase-independent apoptotic pathway during cadmium treatment and that it is located upstream of AIF/Endo G. BNIP-3 silencing inhibits mitochondrial cyt c release to the cytosol and caspase-dependent apoptosis in embryoid body differentiation [57]. BNIP-3 overexpression or recombinant BNIP-3 treatment of isolated mitochondria induce MPT and cyt c release in fibroblasts [48, 58]. We obtained similar results in cadmium-induced rPT cells. Here, the cadmium and Z-VAD-FMK co-treatment group had significantly decreased BNIP-3 protein levels, and apoptosis was significantly prevented, as expected. This proves that the caspase-dependent apoptotic pathway affects the caspase-independent apoptotic pathway. Z-VAD-FMK co-treatment revealed that the two pathways play a similar role (co-promotion or co-suppression), acting synergistically in cadmium-induced rPT cell apoptosis.

The part data was also observed in programmed necrosis induced by Cd. Therefore, we detected level of intracellular ATP and expression level of HMGB1 in the cytoplasm of Cd-treated rPT cells. Mitochondrial ATP production is essential for maintaining ΔΨ and preventing apoptosis [59]. In this study, decreased ATP level (S1 Fig) indicated that abnormal cellular energy metabolism promoted Cd-induced apoptosis in rPT cells; and it was not detected any HMGB1 in the cytoplasm (date not shown). Thus, rPT cells experienced apoptosis rather than programmed necrosis during Cd exposure.

In summary, BNIP-3 acts as an upstream factor in the caspase-independent apoptotic pathway to induce AIF/Endo G translocation. Cadmium activates both the caspase-dependent and caspase-independent apoptotic pathways in rPT cells, and inhibiting one restrains the other. That is, the caspase-dependent and caspase-independent apoptotic pathways are complementary in cadmium-induced rPT cell apoptosis.

## Supporting Information

S1 Fig Effect of Cd on intracellular ATP levels in rPT cells. Cells were treated with Cd (0, 1.25, 2.5 and 5 μmol/L) for 12 h and then collected to measure the cellular ATP levels. Values represent mean ± SEM made in six different primary cultures (n = 6). **P < 0.01 as compared to control.
(TIF)

## Abbreviations

rPT: rat proximal tubular
MPTP: mitochondrial potential translation pore
siRNA: small interfering RNA
BCA: bicinchonininc acid
PI: propidium iodide
ΔΨ: mitochondrial membrane potential
