# Supplementary figures and images for: Caspase-Dependent and Caspase-Independent Pathways Are Involved in Cadmium-Induced Apoptosis in Primary Rat Proximal Tubular Cell Culture
Source: PLoS One. 2016 Nov 18;11(11):e0166823. doi: 10.1371/journal.pone.0166823 (PMC5115828; doi:10.1371/journal.pone.0166823)

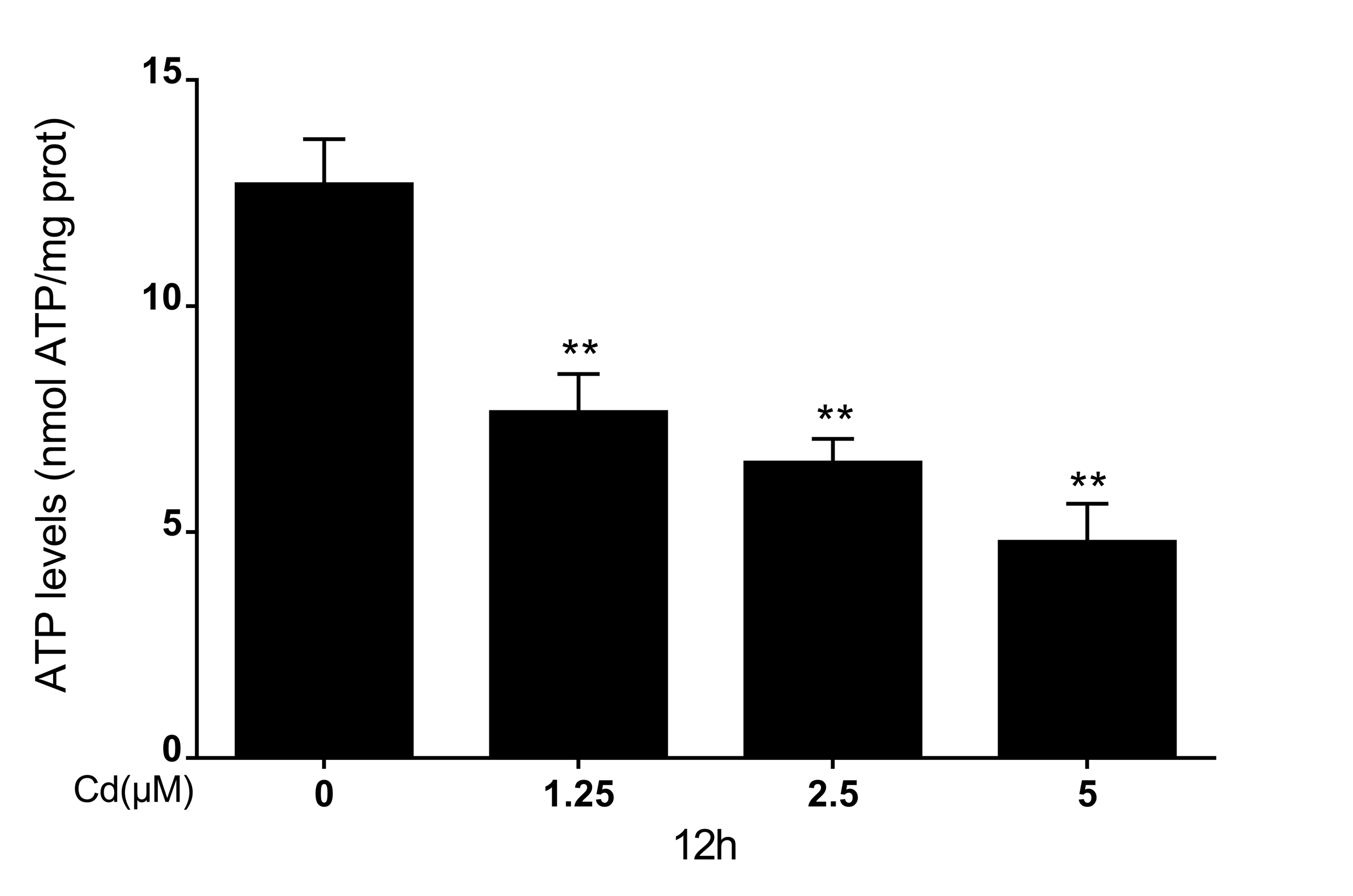

Supplement: S1 Fig — Cells were treated with Cd (0, 1.25, 2.5 and 5 μmol/L) for 12 h and then collected to measure the cellular ATP levels. Values represent mean ± SEM made in six different primary cultures (n = 6). **P < 0.01 as compared to control. (TIF) [file pone.0166823.s001.tif]
